# Supplementary material for: Integrated analysis of the aqueous humor microbiome and lens capsule transcriptome in high myopia cataract: a pilot study
Source: Front Med (Lausanne). 2026 Jun 16;13:1845205. doi: 10.3389/fmed.2026.1845205 (PMC13314463; doi:10.3389/fmed.2026.1845205)
Supplement: Supplementary file 3 [file Data_Sheet_1.zip › 6.PICRUSt2/Meta_Statistic/C372089/Wilcoxon/KEGG_L3.Wilcoxon.pdf]

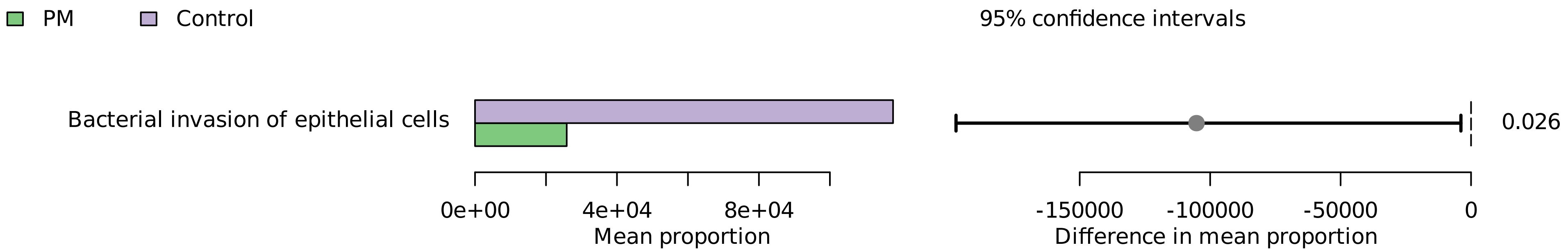

PM
Control
95% confidence intervals
Bacterial invasion of epithelial cells
0.026
0e+00
4e+04
8e+04
Mean proportion
-150000
-100000
-50000
0
Difference in mean proportion
